# The Effect of Vitamin D Supplementation on Rheumatoid Arthritis Patients: A Systematic Review and Meta-Analysis

**DOI:** 10.3389/fmed.2020.596007

**Published:** 2020-10-30

**Authors:** Yuanyuan Guan, Yang Hao, Yun Guan, Huaien Bu, Hongwu Wang

**Affiliations:** ^1^Graduate School, Tianjin University of Traditional Chinese Medicine, Tianjin, China; ^2^Department of Traditional Chinese Medicine, Tianjin First Central Hospital, Tianjin, China; ^3^Crawford School of Public Policy, Asia and Pacific College, Australian National University, Canberra, ACT, Australia; ^4^School of Health Sciences and Engineering, Tianjin University of Traditional Chinese Medicine, Tianjin, China

**Keywords:** vitamin D, rheumatoid arthritis, inflammatory response, meta-analysis, health medicine

## Abstract

**Objective** Observational studies have shown that vitamin D levels are inversely related to rheumatoid arthritis activity, yet evidence from population interventions remains inconsistent.

**Methods:** The PubMed, Cochrane Library, Embase, CNKI, VIP, and Wanfang databases were searched for studies published before June 2020. Information was collected about the pain visual analog scale (VAS), Disease Activity Score 28 (DAS28), serum vitamin D level, tender joint count (TJC), swollen joint count (SJC), erythrocyte sedimentation rate (ESR), C-reactive protein (CRP), and parathyroid hormone (PTH) research data.

**Results:** Six studies (*n* = 438) were included in the meta-analysis. Vitamin D supplementation resulted in a significant improvement in the DAS28 (weighted mean difference (WMD) = −0.41, 95% CI (−0.59, −0.23), *P* < 0.001), ESR (WMD = −3.40, 95% CI (−6.62, −0.18), *P* = 0.04) and TJC (WMD = −1.44, 95% CI (−2.74, −0.14), *P* = 0.03) but not in other outcomes. According to the subgroup analyses, VAS and serum vitamin D were improved in the European ethnic subgroups. TJC and serum vitamin D were improved in the Asian ethnic subgroups. TJC and serum vitamin D were improved in the duration ≤ 12 w subgroups, and the VAS and DAS28 in the duration > 12 w subgroup were different from those of the control group. With a vitamin D dose ≤50,000 IU, only serum vitamin D and TJC improved, and with a vitamin D dose> 50,000 IU, the VAS and DAS28 improved.

**Conclusions:** Compared with placebo control interventions, vitamin D supplementation seemed to be an effective intervention for patients with rheumatoid arthritis. Different doses of vitamin D and durations of intervention produce different effects.

## Introduction

Rheumatoid arthritis (RA) is a chronic autoimmune disease that manifests as a chronic inflammatory response, and persistent synovitis leads to progressive deterioration and impairment of joint function (1). RA bone fragility is caused by systemic inflammation, circulating auto-antibodies and pro-inflammatory cytokine secretion, which together have harmful effects on bone (2). The prevalence of RA is ~1–2% of the world's population, and the prevalence in women is three times that in men (3). Rheumatoid arthritis can occur at any age, but patients around the ages of 40 and 50 are more susceptible. According to the World Health Organization mortality database of 31 countries, RA accounts for almost 18% of all deaths caused by different types of arthritis and other musculoskeletal diseases, but the exact cause of RA remains unknown (4, 5).

Vitamin D (VD) is a fat-soluble hormone that promotes calcium/phosphate metabolism in bones (6). Studies have found that vitamin D has certain effects on other physiological functions and pathological conditions. Specifically, vitamin D has been widely indicated to have an effect on the immune system (7). There is evidence that VD may be involved in rheumatoid arthritis (RA). Studies have found that VD can prevent antigen expression (8) and increase and regulate T cell activity (9).

Higgins et al. (10) found that VD controls the innate and adaptive immune systems mainly through Toll-like receptors (TLRs) and differentiation of T-cells, predominantly Th17 cells, and that these Th17 cells have a crucial role in RA pathology. Aslam et al. (11) found that VD is an important regulator of various genes involved in the immune system. VD mainly prevents the occurrence and development of rheumatoid arthritis by inhibiting cytokine levels (12). In clinical applications, an increasing number of physicians use VD as a supplement to treat RA. The results have a certain effect on improving the symptoms, laboratory indicators or prognosis of RA patients. At the same time, studies on the potential role of VD supplementation in RA treatment have produced inconsistent results. In view of the differences in the current studies, a comprehensive and systematic evaluation of the efficacy of VD as a supplement for RA is necessary.

The aim of this systematic evaluation was to evaluate the efficacy of VD as a supplement for RA compared with a placebo control group and to determine the effects of different VD doses and supplementation times on these patients.

## Methods

### Research Strategy

We searched the PubMed, Cochrane Library, Embase, CNKI, VIP, and Wanfang databases for studies published before June 2020. The search terms were (1) “Vitamin D”[Mesh] OR “Ergocalciferols”[Mesh] OR “Cholecalciferol”[Mesh] OR “Calcifediol”[Mesh] OR “Ergocalciferol” OR “Vitamin D Supplementation” OR “25-hydroxy-vitamin D”; (2) “Arthritis”[Mesh] OR “Rheumatoid Arthritis”[Mesh] OR “Rheumatoid Nodule” OR “Rheumatoid Vasculitis”; and (3) #1 AND #2. The results were filtered for clinical trials and randomized clinical trials. The above search strategy was run in PubMed and was tailored to each database when necessary. Two independent reviewers selected and screened all results, and in cases of disagreement, a third reviewer was asked for advice. The reviewers applied the PRISMA statement guidelines for reporting systematic reviews and meta-analyses (13).

### Eligibility Criteria

The inclusion criteria for this systematic review were as follows: (1) a randomized controlled trial (RCT) design related to the therapeutic effect of VD as a supplement was used; (2) human subjects were recruited; (3) VD supplementation was the main intervention in the experimental group and was compared to a standard treatment control condition; and (4) at least one detail of the outcomes of interest was reported, including changes in serum vitamin D level, tender joint count, swollen joint count, ESR (erythrocyte sedimentation rate), VAS (pain visual analog scale), DAS28 (Disease Activity Score 28), CRP (C-reactive protein), PTH (parathyroid hormone). Data including the mean and standard deviation of each group at baseline and post-intervention, along with the number of participants in each group, were able to be obtained. The exclusion criteria were as follows: (1) duplicate publications; (2) non-intervention designs (such as case-control studies, cohort studies, cross-sectional studies, case reports and experiences, theory research, and reviews); and (3) non-clinical tests and animal experiments.

### Data Extraction

Two review authors independently screened the literature using the predetermined inclusion criteria and extracted the data from the trials. The following information was extracted: participant characteristics, intervention and outcome data, adverse effects, and methodological quality. The reviewers resolved any disagreements about the extracted data from the included studies by consensus and consulted a third review author if the disagreements persisted.

### Risk of Bias Assessment

The risk of study bias was assessed using the Cochrane Handbook for Systematic Reviews. The risk of bias of the studies was evaluated with regard to the following aspects: random sequence generation, hidden methods utilization, blinding method application, incomplete results management, selective reporting of results, and other biases. Funnel diagrams were used to detect publication bias.

### Rating Quality of Evidence

The Grading of Recommendations, Assessment, Development and Evaluation (GRADE) approach was used to rate the quality of evidence for each outcome. The strength of the evidence was categorized as high, moderate, low or very low (14). The GRADE was used to rate the quality of evidence by the consensus of two authors.

### Statistical Analysis

#### Extraction and Merging of Data

The Cochrane Collaboration's Review Manager 5.3 software was used to extract the relevant dichotomous or continuous data from the literature for analysis. Risk ratios (RRs) were calculated for dichotomous data, whereas the mean differences (MDs) and standard deviations (SDs) were calculated for continuous variables. The corresponding 95% confidence intervals (CIs) and forest plots were used in both cases. In our meta-analysis, we used SD values when the data were provided in the same unit. When they were provided in different units, we performed a conversion. The chi-squared and *I*^2^ (inconsistency) tests were used to detect heterogeneity. A *P* < 0.10 or *I*^2^ > 50% indicated that there was significant heterogeneity. The fixed-effect model was used when *P* > 0.10 and *I*^2^ < 50%, and the random-effect model was used when *P* < 0.10 or *I*^2^ ≥ 50%. Subgroup analysis is used to explore the influence of certain characteristics: geographic regions, duration, supplemental dose.

#### Data Conversion

The final DAS28 and VAS values were evaluated as the main results, and serum vitamin D level, TJC, SJC, ESR, CRP, and PTH were evaluated as the secondary indicators. Some trials provided the average value of the indicator but did not provide the SD. We calculated the SD of the indicator using the following formulas: (1) If the number of samples (n) and the standard error (SE) were known, the SD was calculated as:

$$\begin{array}{r} \text{SD}=\text{SE}\times \surd \text{n} \end{array}$$

(2) Estimates of SD were calculated if the number of samples (n), mean, and 95% CI (15, 16) were known, where “a” and “b” are the upper and lower confidence limits, respectively:

$$\begin{array}{r} \text{SD}=\text{a}-\text{mean}/1.96\surd \text{n}\\ \text{SD}=\text{mean}-\text{b}/1.96\surd \text{n} \end{array}$$

## Results

### Study Selection

**Figure 1:**
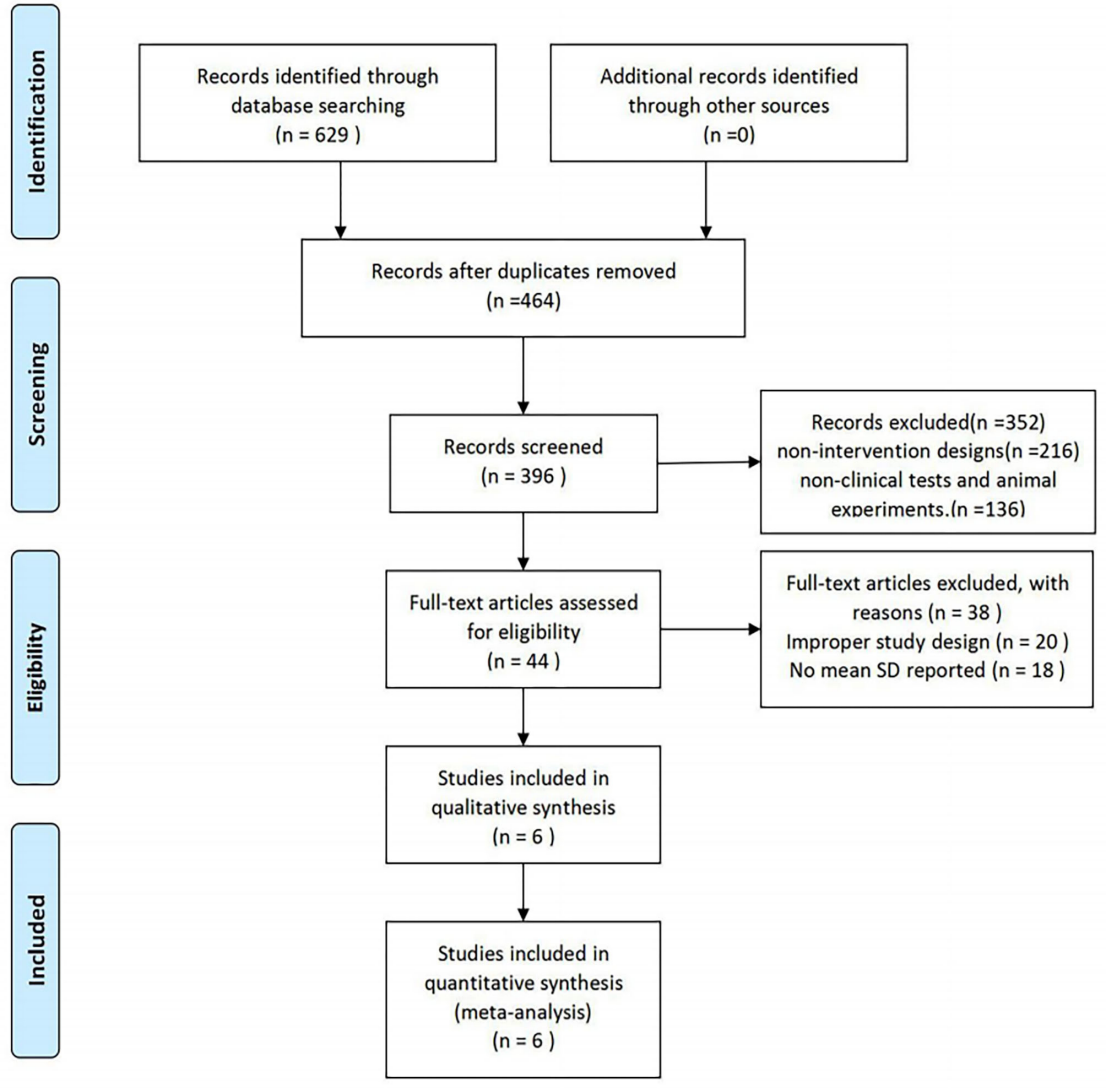
Study selection procedure according to the PRISMA statement (13).

A total of 625 study reports were screened, 165 of which were excluded because they were duplicate publications. After the reading of the titles and abstracts, an additional 352 articles were excluded, and 44 articles were retained. Among them, 38 articles did not meet the inclusion criteria, 20 studies did not have a suitable control group, and 18 studies did not have data that we were able to extract. Finally, six RCTs with a total of 438 participants were included. The PRISMA flow diagram is shown in Figure 1.

### Study Characteristics

The principal study characteristics are summarized in Table 1. Six studies were published between 2011 and 2018. A total of 438 participants were included. The number of participants in the individual studies ranged from 11 to 62. All of the included trials were single-center studies. The included studies were conducted in different geographical regions: North America (19), Europe (20–22) and Asia (17, 18). The duration of the intervention varied from 12 to 24 weeks. All participants had rheumatoid arthritis. Only one trial applied vitamin D and calcium co-supplementation (the same doses of calcium were also given to the control groups) (18). The other studies provided vitamin D supplementation alone.

**Table 1:** Randomized controlled trials included in the systematic review of the effects of vitamin D supplementation on rheumatoid arthritis.

**References**	**Sample**	**Mean age**	**Nation**	**Supplemented**	**Supplementation**	**Time of**	**Outcome**	**Adverse event;**
	**size**	**(T/C)**	**(T/C)**	**dose of vitamin D**		**intervention**	**measured**	**follow-up**
Salesi and Farajzadegan (17)	50/48	49.9 ± 13/50 ± 12.7	Iran	50,000 IU/weekly	Vit D	12 week	①②③④⑤⑥	No
Gopinath and Danda (18)	59/62	44.86 ± 12.33/44.90 ± 12.5	India	500 IU/weekly	Vit D + Ca	12 week	④⑤⑥⑧	No
Hansen et al. (19)	11/11	63 ± 12/53 ± 11	America	50,000 IU/weekly	Vit D	12 week	①⑨	No
Buondonno et al. (20)	18/18	56 ± 14/54 ± 12	Italy	300,000 IU/day	Vit D	12 week	④⑤⑥⑧⑨	No
Adami et al. (21)	51/51	58 ± 13/58 ± 12	Italy	100,000 IU/4 weeks	Vit D	12 week	①②③⑤⑦	No
Soubrier et al. (22)	29/30	59.8 ± 10.9/59.7 ± 8.9	France	100,000 IU once	Vit D	24 week	④⑤⑥⑧	No

*Months were converted to weeks by using 1 mo = 4 wk; years were converted by using 1 y = 52 wk*.

*T, treatment group; C, control group; DAS28, Disease Activity Score 28; VAS, Pain visual analog scale; TJC, Tender joint count; SJC, Swollen joint count; ESR, Erythrocyte sedimentation rate; CRP, C-reactive protein; PTH, Parathyroid hormone*.

*① Serum vitamin D Level; ② Tender joint count; ③ Swollen joint count; ④ ESR; ⑤ VAS (0–100); ⑥ DAS 28; ⑦ DAS 28-CRP; ⑧ CRP; PTH*.

### Quality Assessment

**Figure 2:**
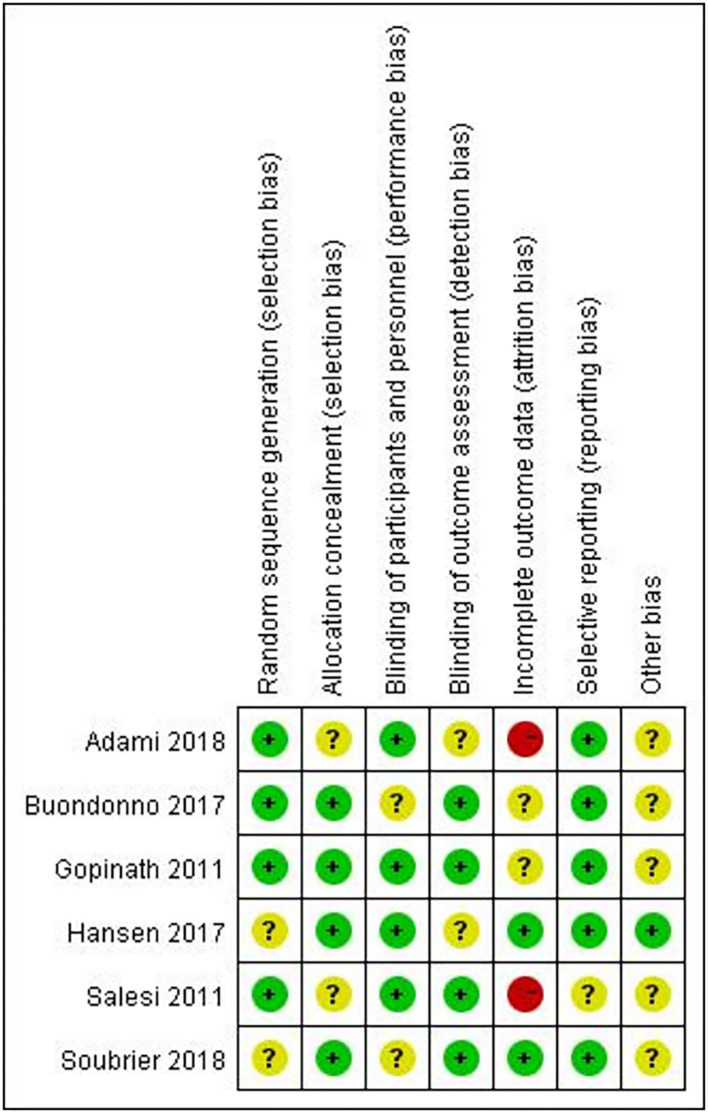
Risk of bias summary: the review authors' judgements about each risk of bias item for each included study.

Figure 2 provides an overview of the risk of bias for the included studies based on the tools provided by the Cochrane Manual. All the included studies used a double-blind approach and reported dropouts. Most of the trials reported allocation concealment and random allocation but did not specify the method used. Four studies (17, 18, 21, 22) reported automatic generation of random sequences by a computer, while two studies (19, 20) reported that the division of participants into an experimental group and a control group with random number tables. Studies that employed selective reporting were unbiased but did include any description to evaluate the existence of other biases. All the included trials reported whether adverse events occurred. It is worth noting that no adverse events occurred in the included trials.

### Study Results

#### The Effect on the VAS

**Figure 3:**
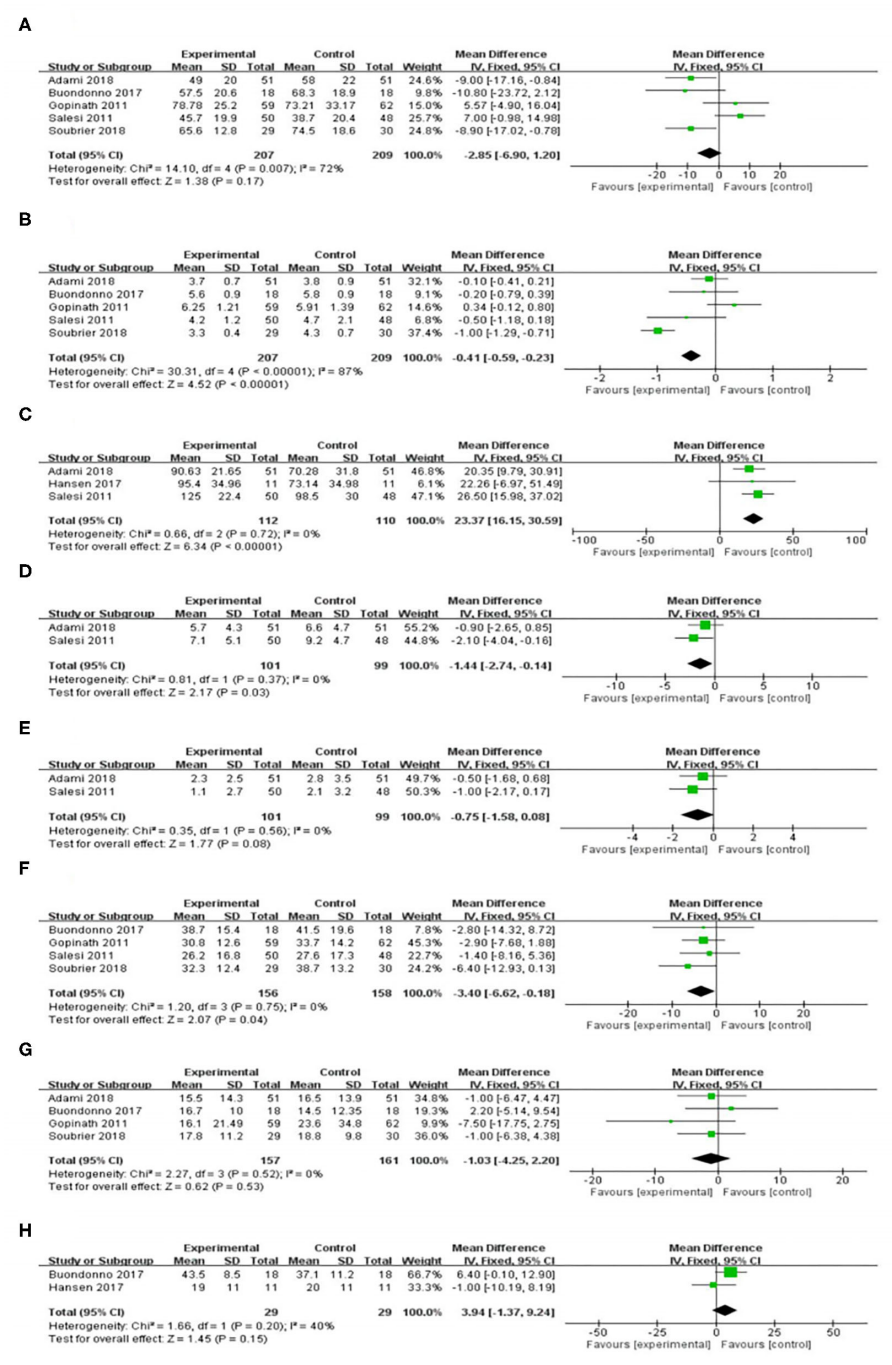
Effect of vitamin D supplementation on **(A)** Patient global pain score; **(B)** Disease Activity Score 28; **(C)** Serum vitamin D level; **(D)** Tender joint count; **(E)** Swollen joint count; **(F)** Erythrocyte sedimentation rate; **(G)** C-reactive protein; and **(H)** Parathyroid hormone.

Five included RCT trials (17, 18, 20–22) with a total of 416 participants provided data on the VAS. There was highly significant heterogeneity among the five studies (*I*^2^ = 72%, *P* = 0.007). The VAS in the vitamin D supplement group was lower than that in the control group, but the difference was not significant [WMD = −2.85, 95% CI (−6.90, 1.20), *P* = 0.17] (Figure 3A). However, vitamin D supplementation produced significant decreases in the following subgroups: Europe, duration > 12 w, and vitamin D dose > 50,000 IU (Table 2).

**Table 2:** Subgroup analyses.

**Subgroups**	**VAS**			**DAS28**			**Serum vitamin D**			**TJC**			**SJC**			**ESR**			**CRP**			**PTH**		
	**WMD**	**95% CI**	***P***	**WMD**	**95% CI**	***P***	**WMD**	**95% CI**	***P***	**WMD**	**95% CI**	***P***	**WMD**	**95% CI**	***P***	**WMD**	**95% CI**	***P***	**WMD**	**95% CI**	***P***	**WMD**	**95% CI**	***P***
**Geographical regions**																								
North America	–	–	–	–	–	–	22.26	[−6.97, 51.49]	0.14	–	–	–	–	–	–				–	–	–	−1.00	[−10.19, 8.19]	0.83
Europe	−9.26	[−14.51, −4.00]	0.0006	−0.54	[−0.74, −0.34]	<0.001	20.35	[9.79, 30.91]	0.0002	−0.90	[−2.65, 0.85]	0.31	−0.50	[−1.68, 0.68]	0.41	−3.25	[−8.94, 2.43]	0.26	−0.31	[−3.71, 3.09]	0.86	6.40	[−0.10, 12.90]	0.05
Asia	6.47	[0.13, 12.82]	0.05	0.07	[−0.31, 0.46]	0.71	26.50	[15.98, 37.02]	<0.001	−2.10	[−4.04, −0.16]	0.03	−1.00	[−2.17, 0.17]	0.10	−2.40	[−6.30, 1.50]	0.23	−7.50	[−17.75, 2.75]	0.15	–	–	–
**Duration**																								
≤ 12 w	−0.86	[−5.53, 3.82]	0.72	−0.06	[−0.28, 0.17]	0.63	23.37	[16.15, 30.59]	<0.001	−1.44	[−2.74, −0.14]	0.03	−0.75	[−1.58, 0.08]	0.08	−2.44	[−6.14, 1.25]	0.20	−1.04	[−5.07, 2.99]	0.61	3.94	[−1.37, 9.24]	0.15
>12 w	−8.90	[−17.02, −0.78]	0.03	−1.00	[−1.29, −0.71]	<0.001	–	–	–	–	–	–	–	–	–	−3.40	[−9.93, 3.13]	0.31	−1.00	[−6.38, 4.38]	0.72	–	–	–
**Vitamin D dose**																								
≤ 50,000 IU	6.47	[0.13, 12.82]	0.05	0.07	[−0.31, 0.46]	0.71	26.01	[16.12, 35.91]	<0.001	−2.10	[−4.04, −0.16]	−0.03	−1.00	[−2.17, 0.17]	0.10	−2.40	[−6.30, 1.50]	0.23	−7.50	[−17.75, 2.75]	0.15	−1.00	[−10.19, 8.19]	0.83
>50,000 IU	−9.26	[−14.51, −4.00]	0.0006	−0.54	[−0.74, −0.34]	<0.001	20.35	[9.79, 30.91]	0.0002	−0.90	[−2.65, 0.85]	0.31	−0.50	[−1.68, 0.68]	0.41	−3.25	[−8.94, 2.43]	0.26	−0.31	[−3.71, 3.09]	0.86	6.40	[−0.10, 12.90]	0.05

#### The Effect on the DAS28

DAS28 levels were reported in five trials (17, 18, 20–22) with a total of 416 participants. There was high heterogeneity among the five studies (*I*^2^ = 87%, *P* < 0.001). There was a significant difference in the DAS28 between the rheumatoid arthritis patients who received vitamin D supplementation and the control group. [WMD = −0.41, 95% CI (−0.59, −0.23), *P* < 0.001] (Figure 3B). Nevertheless, the effects shown in the Europe, duration > 12 w, and vitamin D dose > 50,000 IU subgroups were significantly in favor of the control group (Table 2).

#### The Effect on Serum Vitamin D Levels

Figure 3C shows the forest plot analysis of the effect on serum vitamin D levels. Three RCTs were included (17, 19, 21). There was a significant increase in the serum vitamin D level in the vitamin D supplementation group [WMD = 23.37, 95% CI (16.15, 30.59), *P* < 0.001]. We used a random effects model for the quantitative serum vitamin D level data and showed low heterogeneity (*I*^2^ = 0%, *P* < 0.001). Similar results were demonstrated in the duration ≤ 12 w, Europe and Asia, and vitamin D dose ≤ 50,000 IU or >50,000 IU subgroups (Table 2).

#### The Effect on the TJC

Two trials reported a change in the TJC after the intervention (17, 21). In the comparison of the control and intervention groups, the difference in TJC reduction was significant [WMD = −1.44, 95% CI (−2.74, −0.14), *P* = 0.03] (Figure 3D) and had low heterogeneity (*I*^2^ = 0%, *P* = 0.37). Nevertheless, the effects shown in the Europe and Vitamin D supplement > 50,000 IU subgroups were not significantly different from those of the control group (Table 2).

#### The Effect on the SJC

In terms of reducing the SJC (17, 21), there was no significant difference between the vitamin D supplementation group and the control group [WMD = −0.75, 95% CI (−1.58, 0.08), *P* = 0.08] (Figure 3E). There was low heterogeneity among the included studies (*I*^2^ = 0%, *P* = 0.56). The subgroup analyses suggested similar findings for all subgroups of the intervention (Table 2).

#### The Effect on the ESR

The combined ESR results of the four studies (17, 18, 20, 22) comparing a vitamin D supplementation group with a control group showed significant differences [WMD = −3.40, 95% CI (−6.62, −0.18), *P* = 0.04), with low heterogeneity (*I*^2^ = 0%, *P* = 0.75) (Figure 3F). In the subgroup analysis, the levels in the vitamin D supplementation group were lower than those in the control group, but there was no significant difference (Table 2).

#### The Effect on CRP

Four studies (18, 20–22) reported CRP levels in 318 patients, and 157 of those patients underwent a vitamin D supplementation intervention. The CRP levels decreased in the vitamin D supplementation group compared with the levels in the control group, but there was no significant difference [WMD = −1.03, 95% CI (−4.25, 2.20), *P* = 0.53] (Figure 3G). Similarly, no significant differences were found between the vitamin D intervention group and the control group in the subgroup analysis (Table 2).

#### The Effect on PTH

Two trials (*n* = 58) measured the effect of vitamin D supplementation on PTH (19, 20). Overall, we observed no difference in PTH reduction between the intervention and control groups [WMD = 3.94, 95% CI (−1.37, 9.24), *P* = 0.15]. The heterogeneity was low (*I*^2^ = 40%, *P* = 0.20) (Figure 3H). In the subgroup analysis, it was found that there was a significant difference between the vitamin D supplement group and the control group for the Europe and vitamin D dose> 50,000 IU subgroups (Table 2).

### Publication Bias

**Figure 4:**
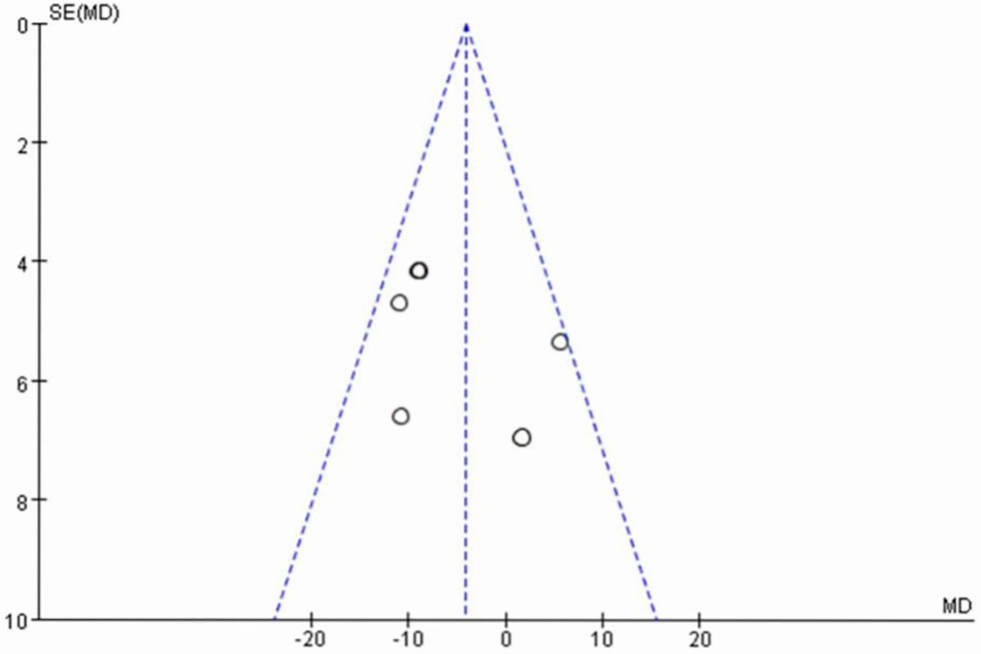
Funnel plot of six randomized controlled trials of vitamin D supplementation for rheumatoid arthritis.

The publication bias of the six RCTs was evaluated with a funnel plot. Figure 4 shows that the publication bias across the studies was small.

### Quality of Evidence

Table 3 presents the quality of evidence by outcome, assessed with the GRADE system. The evidence quality was classified as low for the DAS28, VAS, CRP, and PTH, very low for the SJC, and moderate for the serum vitamin D level, TJC, and ESR.

**Table 3:** Evidence quality rated using the GRADE approach.

**Outcomes**	**No. of studies**	**Limitations**	**Inconsistency**	**Indirectness**	**Imprecision**	**Publication bias**	**Evidence quality**	
DAS28^a^	5	Serious^b^^,^^c^	Serious^d^	Not serious	Not serious	Not found	⊕⊕°°	Low
VAS^a^	5	Serious^b^	Serious^d^	Not serious	Serious^e^	Not found	⊕⊕°°	Low
Serum vitamin D level	3	Serious^b^^,^^c^	Not serious	Not serious	Not serious	Not found	⊕⊕⊕°	Moderate
TJC^a^	2	Serious^b^	Not serious	Not serious	Not serious	Not assessed^f^	⊕⊕⊕°	Moderate
SJC^a^	2	Serious^b^	Not serious	Not serious	Serious^e^	Not assessed^f^	⊕°°°	Very low
ESR^a^	4	Serious^b^	Not serious	Not serious	Not serious	Not found	⊕⊕⊕°	Moderate
CRP^a^	4	Serious^c^	Not serious	Not serious	Serious^e^	Not found	⊕⊕°°	Low
PTH^a^	2	Serious^c^	Serious^d^	Not serious	Serious^e^	Not found	⊕⊕°°	Low

a *DAS28, Disease Activity Score 28; VAS, Patient global pain score; TJC, Tender joint count; SJC, Swollen joint count; ESR, Erythrocyte sedimentation rate; CRP, C-reactive protein; PTH, Parathyroid hormone*.

b *Study had reporting bias*.

c *Study had attrition bias*.

d *Significant heterogeneity was observed in this meta-analysis*.

e *Wide confidence intervals, including values in favor of the experimental group and values in favor of the control group*.

f *Not assessed because a limited number of studies were included in the meta-analyses on TJC and SJC*.

## Discussion and Conclusion

Rheumatoid arthritis is characterized by persistent synovitis, systemic inflammation and autoantibody production (23). Genetic factors and environmental factors are closely related to the incidence of rheumatoid arthritis (24, 25). In developed countries, the prevalence of adult rheumatoid arthritis has increased yearly (26). Active rheumatoid arthritis that does not receive interventional treatment can cause joint damage and even disability (27). Decreased quality of life causes cardiovascular disease and other complications (28, 29). Vitamin D is a fat-soluble hormone that has been extensively studied (30), but recent studies have found that vitamin D has a wide range of immune system activities (31) and that it may be related to the pathogenesis of rheumatoid arthritis (32). Lee et al. conducted a meta-analysis of the sensitivity of vitamin D levels to RA and RA activity in rheumatoid patients, indicating that vitamin D levels were negatively correlated with sensitivity to RA and RA activities (33). There are also studies based on rheumatoid arthritis that suggest that active vitamin D can be used as a parameter for regulating inflammation and that vitamin D has the potential to be a therapeutic biomarker and can even be used to track the disease progression and treatment effect of rheumatoid arthritis patients (34). In previous systematic reviews, no studies have evaluated VD as a supplement for the treatment of RA. In this study, through quantitative synthesis, we found that compared with the control group, RA patients treated with VD as complementary therapy seemed to experience more beneficial effects on the DAS28, TJC, and ESR. In contrast, the VAS, SJC, CRP and PTH showed no benefits. However, although there were no overall beneficial findings, several subgroups showed significant positive effects in terms of demographic characteristics and interventions. Pain is a symptom that rheumatoid arthritis patients urgently need to improve. Previous studies have shown that nearly 90% of patients with rheumatoid arthritis regard pain as the first symptom requiring improvement (35). The VAS score is a commonly used pain score in clinical work and can accurately reflect the degree of pain experienced by patients (36). Based on our combined research results, we found that VD supplementation did not significantly reduce the VAS in rheumatoid arthritis patients. However, in the subgroup analysis, we found that with a vitamin D intervention time> 12 w and vitamin D dose> 50,000 IU, in the VD intervention group, VAS was significantly reduced. Some experimental studies have shown that vitamin D significantly improves nociceptive thresholds and allodynia scores, supporting the analgesic effect of vitamin D on rheumatoid arthritis patients (37). The DAS28 score has important significance as an evaluation indicator of the disease activity of rheumatoid arthritis patients and whether vitamin D is effective after drug treatment (38). Research analysis found that the DAS28 score of patients with rheumatoid arthritis was effectively reduced after vitamin D supplementation. In contrast, when the vitamin D dose was ≤50,000 IU or the duration was ≤12 w, there was no difference between the vitamin D supplement group and the control group. This may be because large-dose and long-term vitamin D supplementation can reduce the inflammatory factor response (39). Mainly through the differentiation of Toll-like receptors and T cells (mainly Th17 cells) to control the innate and adaptive immune system. It can even be used as an important regulator of various genes in the immune system. The SJC, TJC, and ESR are closely related to the DAS28 score (40). Through comprehensive analysis, it was found that the TJC and ESR were significantly reduced in the vitamin D supplement group, which was consistent with the study that found that the DAS28 score was reduced. The ESR subgroup analysis does not support this finding. This may be because the small sample size reduced the reliability of the ESR subgroups. Further research is needed to clarify this connection and explore its potential explanation.

Our study also focused on the effect of vitamin D supplementation on the self-inflammatory response and hormonal immune response of rheumatoid arthritis patients. Therefore, we systematically evaluated the levels of CRP and PTH in rheumatoid arthritis patients and found no significant improvement after VD supplementation. Bjorkman et al. (41) and Moghimi et al. (42) reported results consistent with this finding. CRP concentration is related to bone turnover but not to vitamin D status. Serum vitamin D levels in patients with rheumatoid arthritis may not be related to PTH secretion or activity.

This study has several limitations. First, the overall sample size of this study is limited. Some of the included studies have a small sample size, and the level of evidence defined by GRADE method is not high. Second, most studies included did not assess the effects of sun exposure and dietary intake, and did not indicate whether the patient's rheumatoid arthritis was in the early or late stage, which may have affected the results of the meta-analysis. We conducted a sensitivity analysis of the included RCTs and found that two studies may be the source of most of the heterogeneity. In both studies, the study design was a single-center study with a small number of participants, which may have had an impact on the overall measurement results. In addition, language and publication biases limited our research. Finally, the evaluation included only randomized controlled trials. In the future, more research diversity is needed, such as cooperation between multiple centers, more rigorous clinical reports and prospective research.

We summarize the research status of vitamin D supplementation in patients with rheumatoid arthritis and provide data to support future clinical treatment and trials of rheumatoid arthritis. Although this study shows that supplemental vitamin D can effectively control the DAS28, TJC, and ESR levels of patients with rheumatoid arthritis, the current evidence, potential bias due to the low quality of the research methods and the observed clinical heterogeneity of the examined studies suggest that these findings should be carefully investigated.

## Conclusion

Compared with the control interventions, vitamin D supplementation seemed to be an effective intervention for patients with rheumatoid arthritis. Different doses of vitamin D and the duration of intervention will produce different effects. More RCTs with rigorous research designs are needed to determine the efficacy of vitamin D supplementation in the treatment and improvement of symptoms and inflammatory responses in patients with rheumatoid arthritis and to apply vitamin D supplementation in daily interventions for rheumatoid arthritis patients to improve the symptoms of rheumatoid arthritis and other related chronic diseases.

## Data Availability Statement

All datasets generated for this study are included in the article/Supplementary Material.

## Author Contributions

YuaG, YH, YunG, and HW: conceptualization and methodology. YuaG and YH: data curation and formal analysis. YH and YunG: investigation. HW and HB: project administration and supervision. YuaG: software, writing – original draft, and writing – review and editing. All authors contributed to the article and approved the submitted version.

## Conflict of Interest

The authors declare that the research was conducted in the absence of any commercial or financial relationships that could be construed as a potential conflict of interest.

## Abbreviations

CI: confidence interval
CNKI: China National Knowledge Infrastructure
PubMed: National Library of Medicine
VIP: VIP Database for Chinese Technical Periodicals
MD: mean difference
PRISMA: Preferred Reporting Items Systematic Reviews and Meta-Analyses
RR: risk ratio
RCT: randomized controlled trial
SD: standard deviation
SE: standard error
WMD: weighted mean difference
DAS28: Disease Activity Score 28
VAS: Pain Visual Analog Scale
TJC: Tender joint count
SJC: Swollen joint count
ESR: Erythrocyte sedimentation rate
CRP: C-reactive protein
PTH: Parathyroid hormone.
