# Supplementary material for: The Effect of Vitamin D Supplementation on Rheumatoid Arthritis Patients: A Systematic Review and Meta-Analysis
Source: Front Med (Lausanne). 2020 Oct 30;7:596007. doi: 10.3389/fmed.2020.596007 (PMC7661491; doi:10.3389/fmed.2020.596007)
Supplement: Supplementary file 2 [file Table_2.DOC]

**eTable 1. Search Strategy for Each Database**

| **Database** | **Search strategy** |
| --- | --- |
| Pubmed | #1 ‘‘Vitamin D’’[Mesh] OR ‘‘Ergocalciferols’’[Mesh] OR ‘‘Cholecalciferol’’[Mesh] OR ‘‘Calcifediol’’[Mesh] OR ‘‘Ergocalciferol’’ OR ‘‘Vitamin D Supplementation’’ OR ‘‘25-hydroxy-vitamin D’’  #2‘‘[Arthritis](https://www.ncbi.nlm.nih.gov/mesh/68001168)’’[Mesh] OR ‘‘Rheumatoid Arthritis’’[Mesh] OR ‘‘[Rheumatoid Nodule](https://www.ncbi.nlm.nih.gov/mesh/68012218)’’ OR ‘‘[Rheumatoid Vasculitis](https://www.ncbi.nlm.nih.gov/mesh/68056653)’’  #3 clinical trials[sb] OR randomized clinical trials[ptyp]  #4 #1 or #2  #5 #4 and #3 |
| embase | #1 'Vitamin d'/exp OR 'Vitamin d'  #2 '[Arthritis](https://www.ncbi.nlm.nih.gov/mesh/68001168)'/exp OR Rheumatoid Arthritis  #3 [clinical trials]/lim OR [ randomized clinical trials]/lim  #4 #1 and #2  #5 #4 and #3 |
| Cochrane library | #1 vitamin d:ti,ab,kw (Word variations have been searched)  #2 Rheumatoid Arthritis:ti,ab,kw (Word variations have been searched)  #3 #1 and #2 (restricted as Cochrane clinical trials or randomized clinical trials) |
| CNKI | #1 ‘‘Vitamin D’’[Mesh] OR ‘‘Ergocalciferols’’[Mesh] OR ‘‘Cholecalciferol’’[Mesh] OR ‘‘Calcifediol’’[Mesh] OR ‘‘Ergocalciferol’’ OR ‘‘Vitamin D Supplementation’’ OR ‘‘25-hydroxy-vitamin D’’  #2‘‘[Arthritis](https://www.ncbi.nlm.nih.gov/mesh/68001168)’’[Mesh] OR ‘‘Rheumatoid Arthritis’’[Mesh] OR ‘‘[Rheumatoid Nodule](https://www.ncbi.nlm.nih.gov/mesh/68012218)’’ OR ‘‘[Rheumatoid Vasculitis](https://www.ncbi.nlm.nih.gov/mesh/68056653)’’  #3 clinical trials[sb] OR randomized clinical trials[ptyp]  #4 #1 or #2  #5 #4 and #3 |
| VIP | #1 ‘‘Vitamin D’’[Mesh] OR ‘‘Ergocalciferols’’[Mesh] OR ‘‘Cholecalciferol’’[Mesh] OR ‘‘Calcifediol’’[Mesh] OR ‘‘Ergocalciferol’’ OR ‘‘Vitamin D Supplementation’’ OR ‘‘25-hydroxy-vitamin D’’  #2‘‘[Arthritis](https://www.ncbi.nlm.nih.gov/mesh/68001168)’’[Mesh] OR ‘‘Rheumatoid Arthritis’’[Mesh] OR ‘‘[Rheumatoid Nodule](https://www.ncbi.nlm.nih.gov/mesh/68012218)’’ OR ‘‘[Rheumatoid Vasculitis](https://www.ncbi.nlm.nih.gov/mesh/68056653)’’  #3 clinical trials[sb] OR randomized clinical trials[ptyp]  #4 #1 or #2  #5 #4 and #3 |
| Wanfang databases | #1 ‘‘Vitamin D’’[Mesh] OR ‘‘Ergocalciferols’’[Mesh] OR ‘‘Cholecalciferol’’[Mesh] OR ‘‘Calcifediol’’[Mesh] OR ‘‘Ergocalciferol’’ OR ‘‘Vitamin D Supplementation’’ OR ‘‘25-hydroxy-vitamin D’’  #2‘‘[Arthritis](https://www.ncbi.nlm.nih.gov/mesh/68001168)’’[Mesh] OR ‘‘Rheumatoid Arthritis’’[Mesh] OR ‘‘[Rheumatoid Nodule](https://www.ncbi.nlm.nih.gov/mesh/68012218)’’ OR ‘‘[Rheumatoid Vasculitis](https://www.ncbi.nlm.nih.gov/mesh/68056653)’’  #3 clinical trials[sb] OR randomized clinical trials[ptyp]  #4 #1 or #2  #5 #4 and #3 |
